# Empathic Accuracy: Helpful to Avoid Negative Affect in Old Age?

**DOI:** 10.1093/geroni/igaa057.2142

**Published:** 2020-12-16

**Authors:** Cornelia Wieck, Martin Katzorreck, Denis Gerstorf, Oliver Schilling, Anna Jori Lücke, Ute Kunzmann

**Affiliations:** 1 Leipzig University, Leipzig, Sachsen, Germany; 2 University of Leipzig, Leipzig, Sachsen, Germany; 3 Humboldt University Berlin, Berlin, Berlin, Germany; 4 Heidelberg University, Heidelberg, Baden-Wurttemberg, Germany

## Abstract

Past work suggests age-related declines in empathic accuracy and that these declines may put older people at risk for heightened stress reactivity and low affective well-being. We addressed these questions using data from the fourth wave of the Interdisciplinary Longitudinal Study of Aging (ILSE). To assess empathic accuracy, the young-old (N=115, Mage=63.4, SDage=1.13) and old-old (N=31, Mage=82.3, SDage=.87) participants of ILSE watched six film clips of individuals, who thought-aloud about an emotional autobiographical event, and were asked to rate each individual’s emotions. Subsequently, participants watched a film about Alzheimer’s disease and their subjective and cardiovascular stress reactions were assessed. Empathic accuracy was lower in old-old, as compared with young-old, individuals. Furthermore, empathic accuracy was only associated with low levels of stress reactivity among young-old but not old-old individuals. This suggests that empathic accuracy is not only compromised in very old age, but also appears to be of lower adaptive utility.

